# Lifelog Retrieval From Daily Digital Data: Narrative Review

**DOI:** 10.2196/30517

**Published:** 2022-05-02

**Authors:** Ricardo Ribeiro, Alina Trifan, António J R Neves

**Affiliations:** 1 Institute of Electronics and Informatics Engineering of Aveiro University of Aveiro Aveiro Portugal

**Keywords:** lifelog, lifelogging, information retrieval, image retrieval, computer vision, signal processing, event segmentation, mobile phone

## Abstract

**Background:**

Over the past decade, the wide availability and small size of different types of sensors, together with the decrease in pricing, have allowed the acquisition of a substantial amount of data about a person’s life in real time. These sensors can be incorporated into personal electronic devices available at a reasonable cost, such as smartphones and small wearable devices. They allow the acquisition of images, audio, location, physical activity, and physiological signals among other data. With these data, usually denoted as lifelog data, we can then analyze and understand personal experiences and behaviors. This process is called lifelogging.

**Objective:**

The objective of this paper was to present a narrative review of the existing literature about lifelogging over the past decade. To achieve this goal, we analyzed lifelogging applications used to retrieve relevant information from daily digital data, some of them with the purpose of monitoring and assisting people with memory issues and others designed for memory augmentation. We aimed for this review to be used by researchers to obtain a broad idea of the type of data used, methodologies, and applications available in this research field.

**Methods:**

We followed a narrative review methodology to conduct a comprehensive search for relevant publications in Google Scholar and Scopus databases using lifelog topic–related keywords. A total of 411 publications were retrieved and screened. Of these 411 publications, 114 (27.7%) publications were fully reviewed. In addition, 30 publications were manually included based on our bibliographical knowledge of this research field.

**Results:**

From the 144 reviewed publications, a total of 113 (78.5%) were selected and included in this narrative review based on content analysis. The findings of this narrative review suggest that lifelogs are prone to become powerful tools to retrieve memories or increase knowledge about an individual’s experiences or behaviors. Several computational tools are already available for a considerable range of applications. These tools use multimodal data of different natures, with visual lifelogs being one of the most used and rich sources of information. Different approaches and algorithms to process these data are currently in use, as this review will unravel. Moreover, we identified several open questions and possible lines of investigation in lifelogging.

**Conclusions:**

The use of personal lifelogs can be beneficial to improve the quality of our life, as they can serve as tools for memory augmentation or for providing support to people with memory issues. Through the acquisition and analysis of lifelog data, lifelogging systems can create digital memories that can be potentially used as surrogate memory. Through this narrative review, we understand that contextual information can be extracted from lifelogs, which provides an understanding of the daily life of a person based on events, experiences, and behaviors.

## Introduction

### Background

With the expansive use of technology by humans, such as smartphones and wearable devices accessible worldwide, the acquisition of data about a person’s activity is changing dramatically, allowing the acquisition of a huge amount of different types of data every day in the form of images, locations, and physiological signals. With the rapid development of Internet of Things solutions, these personal data can be applied in a wide range of applications. One such application is lifelogging.

Lifelogging is defined as a form of pervasive computing, consisting of a unified digital record of the totality of an individual’s experiences, which is usually called a lifelogger, captured multimodally through digital sensors and stored permanently as a personal multimedia archive. In a simple way, lifelogging is the process of tracking and recording personal data created through our activities and behavior [1,2].

The idea of storing knowledge and information to provide an auxiliary memory to support people was envisioned by Vannevar Bush [3]. At the end of the Second World War in 1945, Vannevar Bush presented the Memex concept to the world. Memex represented a device in which an individual stores knowledge and information, such as his books, records, and communications, based on association, similar to the brain, and exposes it as a memory aid. Bush also envisioned 2 other devices that have come to life: the minicamera worn on the forehead that would allow users to take photographs from their point of view and a device that would record voice in text format. Remarkably, the use of these 3 devices together would enable what could be considered as the starting point of lifelogging.

With the evolution of digital technologies over the years, solutions to record, store, and organize a lifetime of information and knowledge have become possible, as envisioned by Vannevar Bush. Bush’s vision remains an inspiration for many information retrieval and lifelogging systems. However, the amount of information available to be stored and processed today is difficult to analyze and retrieve. To overcome this problem, a wide range of research fields can be explored, such as image and information retrieval, knowledge extraction, image understanding, sentiment analysis, and data mining just to name a few, which provide solutions to organize, process, and retrieve personal data. These personal data are also named as lifelogs and can be used as surrogate memory within a lifelogging system capable of organizing and managing these lifelogs [2]. Therefore, the extraction of relevant information from personal lifelogs can be used to improve the quality of everyday life for people with memory problems or even used as a digital diary.

The practice of lifelogging has become an important resource of contextual data. Projects such as Digital Eye Glass [4-11], MyLifeBits [12-16], and SenseCam [17,18] were the most relevant in the past. The amount of lifelog data (volume), the different types of data obtained from several sources (variety), and the agility to process the lifelogs and generate the necessary information (velocity) make lifelogging an interesting and challenging big data application [2,19]. For example, Gurrin et al [20] started to analyze the large visual lifelogs that were captured during a period of more than a year. Therefore, it is not surprising that the complexity and interdisciplinary challenges are increasing the attention on the lifelogging subject from the research community.

### Objectives

Memory is often compared with a computer as it constitutes an information processing system. Both systems have basic functions such as encoding (input and processing of information), storage (retention of information), and retrieval (obtaining information from the storage) [21]. The loss of information from memory, also known as forgetting, occurs when a failure in encoding occurs owing to interference or other memory errors. Encoding failures can be circumvented through lifelogging. Lifelogs, particularly visual lifelogs, provide context cues that can help recall and recognition [21]. As a result, lifelogging has the potential for supporting memory augmentation, which can be applied to aid memory retrieval not only for people with dementia but also for healthy people.

The world’s population above the age of 60 years has been increasing since 1950, and it is estimated to reach approximately 2.1 billion by 2050 [22]. Consequently, the World Health Organization recognized dementia as a public health priority and proposed a global action plan with several action areas, which includes the development, implementation, and improvement of surveillance and monitoring systems, to improve the functional trajectories of people with dementia, their careers, and families [23]. Considering everything mentioned above, patients with dementia could benefit from a lifelogging application that would work as a digital everyday life journal or as a personal historical record [24].

Lifelogging technologies give us the opportunity to create human digital memories, allowing us to represent and understand every moment of our lives and store this information for further use. However, each memory has specific cues, which can be captured from multiple sources based on our surroundings, such as visual cues, verbal and environmental sounds, locations, and actions, thus providing a large amount of contextual information that requires an interactive software tool to retrieve and explore the memory space. In this narrative review, we have discussed about the several types of personal lifelogs and lifelogging applications used to retrieve these lifelogs.

## Methods

### Search Strategy

This narrative review [25,26] explored a broad perspective of lifelogging approaches and technologies with the aim of synthesizing and understanding the literature on this research topic. Google Scholar and Scopus databases were used to conduct an iterative search based on a combination of search terms or keywords and appropriated Boolean operators to identify relevant publications.

The following search terms were explored: *(lifelog OR lifelogging) AND (visual OR audio OR location OR physical activity OR physiological signal OR dementia)*. A search period was included for searching the publications within the period of 2008 to 2020. However, to explore a historical view of the research topic, relevant publications before 2008 were manually identified and included. This additional inclusion of potential manuscripts of interest was based on our knowledge of this research topic and the association of authors and references of the publications included previously. Only publications in English were considered.

### Inclusion and Exclusion Criteria

A total of 411 search results were screened based on the relevance of their title and abstract. Of these 411 publications, 114 (27.7%) publications were selected for full-text analysis. Of the 114 publications, 31 (27.2%) publications were excluded based on their content, and finally, 113 publications were included in our narrative review after including several other publications through citation searching.

**Figure 1:**
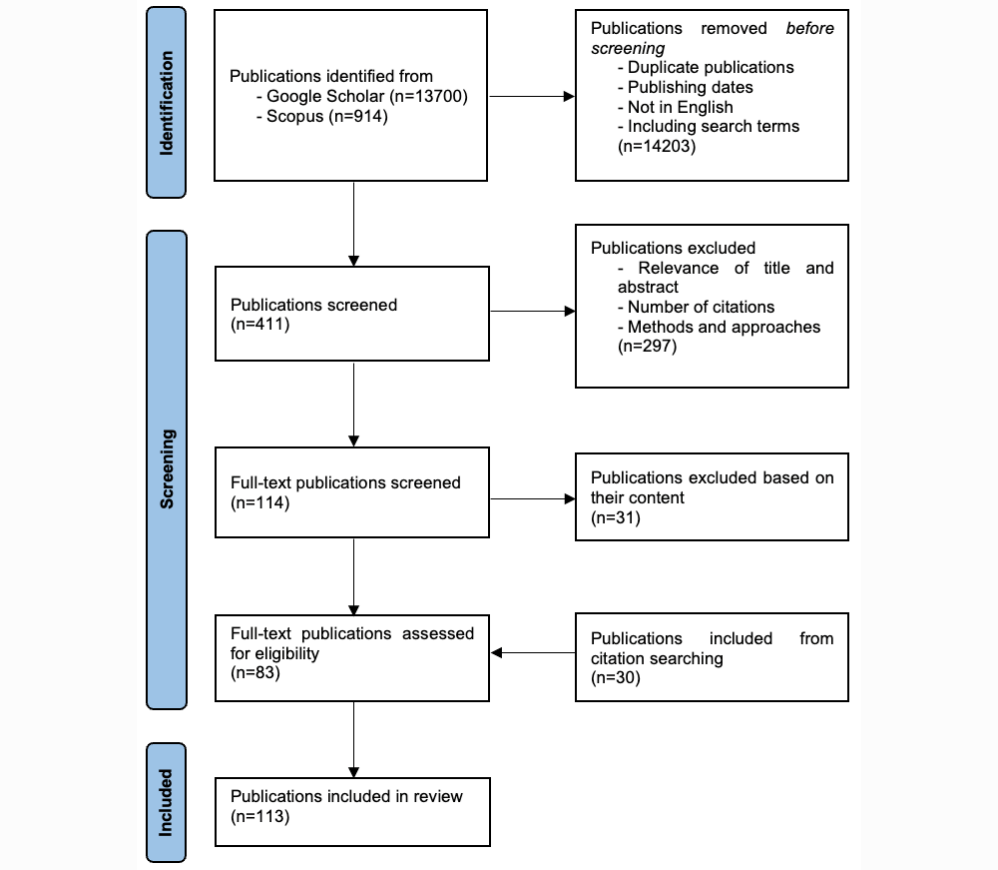
Flow diagram of the literature selection process for this narrative review.

Figure 1 shows a flow diagram with the search strategy that led to the included citations, following the PRISMA (Preferred Reporting Items for Systematic Reviews and Meta-Analyses) 2020 guidelines [27]. Initially, our search resulted in a total of 14,614 articles by searching the keywords *lifelog* and *lifelogging*. However, as the number of resulting publications was high, we chose to combine keywords, such as *visual*, *audio*, *location*, *physical activity*, *physiological signs*, and *dementia*. Several duplicate articles were excluded, and we selected 2.81% (411/14,614) of the publications. To further restrict our article selection, several articles were excluded based on the relevance of their title and abstract, number of citations, relevance, and approaches or methods. As a result of this search, 27.7% (114/411) of the publications were selected and fully reviewed. Moreover, 27.2% (31/114) of these publications were excluded based on their content. Finally, based on our knowledge of this research topic and by exploring the publication records of the authors of the selected papers, we included 30 more articles to conclude our manuscript collection process with 113 publications.

## Results

### Types of Lifelog Data

#### Overview

**Figure 2:**
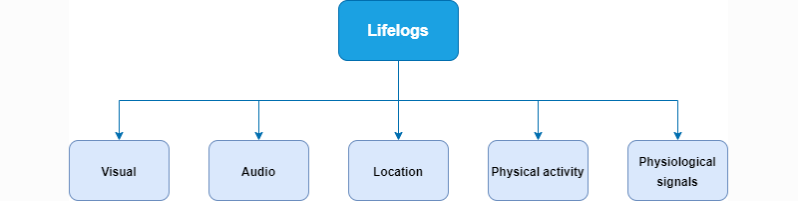
Main types of lifelog data used in lifelogging identified from our review of the literature.

Recent technological advances have introduced new types of sensors and devices that allow the compilation of vast archives of personal data. According to our research, the review of the literature proposes that the most prominent data explored in the lifelogging research are images, videos, locations, physical activities, and physiological signals, as presented graphically in Figure 2. In visual lifelogs, data are captured by cameras in the form of images or videos. Although audio is not widely used, the voice of the users or sounds in the environment can be useful data that can be integrated into lifelogging systems. The locations can be understood in 2 different ways, such as GPS locations (longitude and latitude) or physical locations (University of Aveiro, home, work, etc). Currently, devices such as smartwatches, which are wearable devices that incorporate sensors such as accelerometers, gyroscopes, force sensors, and pressure sensors, are frequently used by many people. They enable the extraction of information to monitor physical activities. However, these types of wearable devices also incorporate other sensors capable of recording physiological signals such as heart rate and body temperature.

Table 1 summarizes the types of data used in the selected studies on lifelogging. Description of the several approaches is presented in the following subsections. As seen in Table 1, visual data are the most used owing to its richness and the advances in image processing algorithms that allow the extraction of relevant information from images or video. However, several studies have already been reported on the use of other types of data and multimodal solutions.

**Table 1:** Studies and types of data used.

Study	Visual	Audio	Location	Physical activity	Physiological signals
Piasek et al [24]	✓ (SenseCam)	—^a^	—	—	—
Hodges et al [17]	✓ (SenseCam)	—	—	—	—
Doherty et al [28,29]	✓ (SenseCam)	—	—	—	—
Gurrin et al [30]	✓ (smartphone)	—	—	—	—
Pauly-Takacs et al [31]	✓ (SenseCam)	—	—	—	—
Wang et al [32,33]	✓ (SenseCam)	—	—	—	—
Song et al [34]	✓ (Google Glass)	—	—	—	—
Li et al [35]	✓ (SenseCam)	—	—	—	—
Bolanos et al [36]	✓ (Narrative Clip)	—	—	—	—
Talavera et al [37]	✓ (Narrative Clip and SenseCam)	—	—	—	—
Dimiccoli et al [38]	✓ (data sets)	—	—	—	—
Gupta and Gurrin [39]	✓ (OMG Autographer)	—	—	—	—
Fan et al [40]	✓ (Narrative Clip)	—	—	—	—
Garcia del Molino et al [41]	✓ (data sets)	—	—	—	—
Furnari et al [42]	✓ (Looxcie LX2)	—	✓ (semantic)	—	—
Oliveira-Barra et al [43]	✓ (data sets)	—	—	—	—
Ellis and Lee [44]	—	✓	—	—	—
Shaikh et al [45]	—	✓	—	—	—
Shah et al [46]	—	✓	✓ (GPS)	—	—
Yamano and Itou [47]	—	✓	✓ (GPS)	—	—
Ziaei et al [48]	—	✓		—	—
Li et al [49]	—	—	✓ (GPS)	—	—
Tanaka et al [50]	—	—	✓ (GPS)	—	—
Aizawa et al [51], Hori et al [52], and Datchakorn et al [53]	✓	✓	✓ (GPS and semantic)	✓ (multiple sensors)	—
Doherty et al [54]	✓ (SenseCam)	—	—	✓ (smartphone)	—
Hurvitz et al [55]	—	—	✓ (GPS and semantic)	✓ (multiple sensors)	—
Yang et al [56,57]	—	—	—	✓ (multiple sensors)	—
Dobbins et al [58]	—	—	—	✓ (data sets)	✓ (data sets)
Ni et al [59]	—	—	✓ (GPS)	✓ (smartphone)	—
Kim et al [60]	—	—	—	—	✓ (smartwatch)
Choi et al [61]	—	—	—	—	✓ (multiple sensors)
Dobbins and Fairclough [62]	—	—	—	—	✓ (multiple sensors)

^a^The study does not use this type of data.

#### Visual

We can observe from Table 1 that several studies on lifelogs have explored the use of visual data. Visual lifelogs are generally collected in the form of photographic or videographic records to trigger memories. Photographs are the preferable representation of autobiographical memories [63,64]. In recent years, wearable devices capable of capturing images or videos continuously from a personal perspective are increasingly used. Examples of these wearable devices are SenseCam, OMG Autographer, Narrative Clip, Google Glass, and GoPro. In addition to these wearable devices, smartphones with high-quality cameras and other sensors are also an important tool for lifelogging. Gurrin et al [30] explored the use of smartphones as an alternative solution to wearable devices such as SenseCam and described several advantages of using smartphones as behavior monitoring devices.

The ability of devices, such as SenseCam, to improve autobiographical memory was studied on a patient with amnesia [17]. This case study indicated that short-term recall improved owing to the use of SenseCam. Furthermore, the use of SenseCam also enhanced long-term memory of autobiographical events. In contrast to the SenseCam application, the written diary helped to recall events in the short term, but not in the long term. The main challenge that this type of devices, and consequently visual lifelogs, face is the processing of such tremendous amounts of data [18]. It is essential to develop techniques that are able to automatically label, segment, and present relevant images in a meaningful sequence.

Pauly-Takacs et al [31] used the images captured by SenseCam during a walk to assist a boy aged 13 years, with profound episodic memory difficulties in remembering those moments. The result of this experiment shows the ability of the images to support the formation of personal semantic memories and memory rehabilitation. In addition to helping in the improvement of retrospective memory, SenseCam can also be applied to patients with dementia, but as a cognitive stimulation therapy. A case study was conducted with the aim of mentally stimulating the patient and encouraging factual and opinionated communication [24].

It is obvious that visual lifelogs are essential as memory reminders to reconstruct previous life experiences, but these lifelogs can be used in other use cases, such as general lifestyle analysis. Doherty et al [28,29] proposed a method to automatically classify visual lifelogs into different lifestyle traits using images collected by SenseCam. The camera captures details of the individual’s everyday activities, in an approach to build a memory of the past. Moreover, Doherty et al [54] used SenseCam images to complement accelerometry measures to identify behavior type and context information across a range of activity episodes.

It is essential to develop techniques that are capable of summarizing the large number of images collected through visual lifelogging. Similarly, Wang and Smeaton [32] proposed a technique for identifying everyday activities captured using SenseCam. It is worth noting that these findings are consistent with previous literature [2,21]. In terms of daily human activities, a very wide range of semantic concepts can be identified in visual lifelogs. For the same activity, a variety of semantic concepts can be observed across individuals. Wang et al [33] characterized everyday activities and behaviors of individuals based on the detection of semantic concepts that appear in visual lifelogs obtained from events that have been automatically segmented based on the technique introduced in the study by Lee et al [65].

In another study conducted using SenseCam, a day of a user was recorded by taking a photo every 30 seconds [35]. Following the lifelogging process, the user reviewed the collected data and classified the day into 12 events to create a ground truth. This method has the potential to retrieve autobiographical events, enabled by the creation of visual lifelogs. Therefore, the use of a wearable camera along with the methods mentioned in this paper constitutes a promising approach to help people retrieve their memories.

In the study by Song et al [34], several egocentric videos were recorded using Google Glass, which captured the diversity and complexity of different daily human activities from a first-person perspective. These videos were collected from 10 different individuals and contained 13 categories of activities relevant to lifelogging applications. Song et al [34] performed several experiments through which they accurately recognized these activities by adopting the dense trajectory approach.

Bolanos et al [36] proposed a method for creation of visual summaries of a set of egocentric images captured by a wearable camera, the Narrative Clip. This summarization aims to support people with neuronal degradation. Other similar studies have been proposed based on the same methodology of clustering-based event segmentation [37] and summarization using contextual and semantic information [38].

Recently, methods based on deep learning to extract visual concepts from images have grown rapidly, making it possible to automatically extract and annotate visual lifelogs accurately. Gupta and Gurrin [39] proposed event segmentation of visual lifelogs based on 2 different approaches for visual concept extraction and image classification, such as objects and activities. The visual lifelogs were collected using a wearable camera, OMG Autographer.

Fan et al [40] proposed the compilation of a journal using the captions of photo streams acquired through camera-based lifelogs. This type of lifelogging collects a large number of images, which in turn are of low quality, noisy, and ambiguous, as they are taken automatically. In this study, 2 authors used Narrative Clip cameras for 5 months to create a data set.

Most studies that used visual lifelogs collected images or videos and created data sets that often contain very limited data, which results in insufficient data to train machine and deep learning algorithms efficiently. In the study by Garcia del Molino et al [41], a large-scale data set with a first-person perspective was created with >1.5 million images captured by 57 users using a wearable camera to train a visual context predictor. This approach can be used to model daily activities and learn the associations between different scenes.

Furnari et al [42] presented a method for temporal segmentation based on personal locations. This study is very promising because it achieves results that are as accurate as those of other methods in the literature. Oliveira-Barra et al [43] proposed a comprehensive methodology for egocentric photo stream analysis. They performed a summary of autobiographical episodes and a semantic key-frame selection and, finally, implemented text-based inverted index retrieval techniques. The episode temporal segmentation was based on semantic regularized–clustering [38]. This model was applied to a data set, and the results suggest that this system stimulates the memory of patients with mild cognitive impairment; for example, patients with dementia.

#### Audio

As stated in Table 1, a lifelogging application can also use audio lifelogs, generally captured by wearable audio recorders, smartphones, or video cameras that can record audio for several hours or days using a microphone. In the MyLifeBits project [12,13], Gordon Bell used a wearable microphone to record audio clips and stored them in his personal lifelogs. Ellis and Lee [44] described several practical advantages of using audio lifelogs and conducted experiments with different equipment and techniques. Totally, there are 3 major advantages of using audio lifelogs [44]: audio devices, such as microphones, are less sensitive to positioning or motion than cameras; audio data are smaller in file size than videos or image sequences; and audio archives can provide a wide range of useful information, such as location, activities, people, and words.

Audio lifelogs can provide useful information to lifelogging systems, and human activities are reflected in a rich variety of acoustic events and environmental sound cues. Shaikh et al [45] proposed a method to detect and classify activities of daily living, such as laughing, talking, cooking, and so on, and location of the person, such as inside a train, at home, at school, and so on, from the environmental sound cues. Shah et al [46] proposed a lifelogging system using audio records that included speech, music, and environmental sounds. In large audio lifelogs, manual browsing and searching for events or specific audio clips is time-consuming. Therefore, to deal with several types of audio and build an easy, intuitive, and efficient lifelogging application, a generalized and more complex approach was presented in the study by Shah et al [46].

Other studies have used audio lifelogs to segment and classify them according to several characteristics. For example, Yamano and Itou [47] recorded audio lifelogs using wearable microphones and conducted several experiments that enabled browsing these lifelogs. The audio lifelogs were segmented and clustered into events to classify them according to place, speaker, and time. Ziaei et al [48] proposed an analysis system, which automatically estimates the number of unique people and environments using personal audio records.

#### Location

Lifelogs based on locations can be recognized in 2 different ways: GPS coordinates, such as longitude and latitude, and physical or semantic locations characterized by the place or environment, such as home, office, or more specific locations such as the University of Aveiro. Literature indicates that GPS tracking devices and wearable devices improve the users’ self-esteem when evaluating the effects on the quality of life [66,67]. It is important to note that in this case, the data from GPS tracking devices were not intended to retrieve memories. Nevertheless, the location information may complement visual lifelogs by identifying where the images were taken. This information is usually expressed as coordinates. Moreover, lifelogs offer the option to register relevant locations under intuitive names such as *my son’s house* [42,49]. When the user checks her lifelog, both the image and the location are displayed. Thus, the user may recall the corresponding memory more easily, even if no spatial cues are visible in the image.

Li et al [49] proposed a method for relating user activities to their location. The authors used spatial and temporal constraints to infer where the user worked or studied. Although this method does not correctly identify all the activities, the results are promising. Furthermore, the proposed method points to the possible automatic compilation of a journal with the places and activities of everyday life by just using a smartphone, which, in turn, can aid memory retrieval.

In the study by Tanaka et al [50], a method for daily context recognition by recording lifelogs based on GPS location from a smartphone was proposed. The proposed method recognizes the lifelogger’s location and activity as contexts. It can also recognize several contexts at the same location; for example, in a shopping mall, the method can distinguish between shopping, eating a meal, or watching a movie at the cinema. By using a smartphone, the lifeloggers can track their activities over time and observe their daily life in more detail.

#### Physical Activity

Physical activity is fundamental for human beings and is associated with better general health status and improved quality of life. Accelerometers, gyroscopes, goniometers, force sensors, and pressure sensors enable the collection of diverse information. When strategically placed on the user, these sensors can assess the gait and detect falls [68]. Moreover, these sensors are often incorporated into smartwatches or smart bands to monitor physical activity [40]. In addition to counting steps and estimating walked distance, smartwatches and wristbands can record the heart rate and detect stair climbing, arousal, stress, and excitement through electrodermal activity [21].

Doherty et al [54], following their previous study on event-based segmentation [28] and recognition of human activities [29], proposed the use of accelerometers combined with images from wearable cameras to identify certain physical activity behaviors. In this approach, the accelerometer data determined the event boundaries, and the authors could identify sedentary and light, moderate, and vigorous intensity physical activities.

With the easy accessibility of sensors such as accelerometers, which measure the acceleration forces acting on an object or person to determine the object’s position in space and monitor the movement, Hurvitz et al [55] proposed methods to measure and analyze activity behaviors using data, such as location, activity, and environment, collected from the combination of accelerometers, GPS data, and travel diaries. The authors also provided an interface tool to structure and visualize location and physical activity data simultaneously.

Yang et al [56,57] studied several existing lifelogging physical activity measurement devices and identified some measurement uncertainties in an Internet of Things environment that impact the efficiency and accuracy of lifelogging and health applications.

Several diseases such as obesity, hypertension, and cardiovascular diseases are correlated with insufficient physical activity. Dobbins et al [58] proposed an approach to collect and process data from triaxial accelerometers and a heart rate monitor to classify physical activities, such as lying, sitting, running, working on computer, and walking, into different activity levels. In addition to this classification, a visual interface was provided to display the classification of daily physical activities of the user on a smartwatch.

Recently, Ni et al [59] explored a 2-stage hybrid model to predict human physical activity status from lifelogging data collected by wearable sensors. Their goal was to provide health care analytics to support individual decisions in real-time monitoring and statistical analysis, provide personalized advice to individuals, and ultimately, encourage positive attitudes toward healthy lifestyles.

#### Physiological Signals

**Figure 3:**
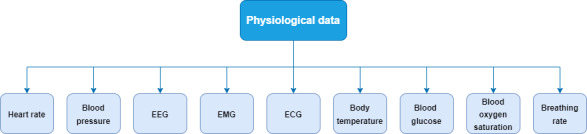
Summary of the main physiological data. ECG: electrocardiogram; EEG: electroencephalogram; EMG: electromyogram.

Physiological data are inevitably related to the health care service area. These data have been increasingly used in lifelogs over the years, which can be explained by the expansion of the fitness industry [21,60]. The main physiological data are presented in Figure 3. The most relevant data are heart rate, blood pressure, electroencephalogram, electromyogram, electrocardiogram, blood oxygen saturation, blood glucose, body temperature, and breathing rate [61,68,69]. However, the sensors needed to collect most of these data still have to be incorporated into more practical devices before they become prominent in lifelogging applications.

Heart rate is related to user activity; therefore, it plays a relevant role; for example, when the intention is to identify user activities from visual lifelogs. In the study by Dobbins et al [58], the use of heart rate information was combined with an accelerometer to detect physical activity and support people with diseases such as obesity. Another relevant biological signal is blood pressure, and similar to heart rate, the respective sensors can be incorporated into wearable devices, particularly smartwatches [69].

Dobbins and Fairclough [62] collected lifelogging data from multiple sources including physiological signals, such as ECC and photoplethysmogram data, and driving data, such as the speed of the vehicle, location, and first-person environment images, to develop several classifiers for detecting stress in real-world driving.

### Challenges and Data Sets

Over the past years, the term *lifelogging* has received significant attention from both research and commercial communities. The events that introduced the lifelogging concept to the academic community were the Association for Computing Machinery Continuous Archiving of Personal Experiences workshops from 2004 to 2006 [70-72]. These workshops were essential for those who previously designed and developed solutions independently [6,44,73,74], share tools and experiences, and lead lifelogging as an emerging research area.

Table 2 presents the most relevant data sets existing in the literature for lifelog research.

**Table 2:** Data sets.

Data sets	Visual	Audio	Location	Physical activity	Physiological signals
NTCIR^a^-12 lifelog [75]	✓ (OMG Autographer)	—^b^	✓ (semantic)	✓ (smartphone)	—
NTCIR-13 lifelog [76]	✓ (Narrative Clip)	✓ (music listened)	✓ (semantic)	✓ (smartphone)	✓ (multiple sensors)
NTCIR-14 lifelog [77]	✓ (OMG Autographer)	✓ (music listened)	✓ (semantic)	✓ (smartphone)	✓ (multiple sensors)
EDUB^c^ [78]	✓ (Narrative Clip)	—	—	—	—

^a^NTCIR: National Institute of Informatics Testbeds and Community for Information Access Research.

^b^The data set does not contain this type of data.

^c^EDUB: Egocentric Dataset of the University of Barcelona.

In 2016, the first test collection for personal lifelog data was introduced [75], which has been used for the National Institute of Informatics Testbeds and Community for Information Access Research (NTCIR)-12–Lifelog task [79]. It promoted a comparative evaluation of information access and retrieval systems operating over personal lifelogs. The lifelogs in this test collection consisted of images from 3 lifeloggers using the wearable camera, OMG Autographer. It also contained several semantic locations, such as home, work, and so on, and physical activities, such as walking, transport, and running. The data set was anonymized to ensure the privacy of both lifeloggers and individuals by removing identifiable content, such as recognizable faces and absolute GPS locations. The test collection also included a set of topic descriptions, which represent the retrieval and reflection reason of the lifelogger for accessing memories [75].

Consequently, challenges regarding lifelogging started emerging. The First Workshop on Lifelogging Tools and Applications in 2016 [80] aimed to discuss approaches to lifelog data capture, analytics, and applications, thus identifying opportunities and challenges for researchers in this new and challenging area. In 2017, the Second Workshop on Lifelogging Tools and Applications was organized simultaneously with the lifelog evaluation tasks, NTCIR-13 Lifelog-2 Task [76] and ImageCLEFlifelog 2017 Task [81].

The ImageCLEFlifelog 2017 challenge was introduced with the NTCIR-12–Lifelog data set [75], but different subtasks were proposed to the participants. However, in the NTCIR-13 Lifelog-2 Task, the organizers created a new data set based on the requirements of the first test collection for personal lifelog data. In addition to the tasks of NTCIR-12–Lifelog, they addressed 2 different challenges for lifelog data organization and retrieval [76].

Since then, workshops and tasks have been organized to advance research on some of the key challenges: ImageCLEFlifelog challenges [82-84]; Lifelog Search Challenge [85-87], which aims to encourage the development of efficient interactive lifelog retrieval systems; and NTCIR Lifelog Tasks [77]. Over the years, these challenges have focused on creating a comparative benchmark activity for lifelogging applications, and data sets used in each of them are very similar or even the same. These data sets start with the first test collection for personal lifelog data [75], which they extend or improve.

In addition to the data sets used in these challenges, several other data sets containing egocentric data are available [34,42,78,88]. However, most of these data sets focus on different and smaller amounts of data for specific use case applications and not on capturing all the daily activities and behaviors of a lifelogger. An example of these data sets is the Egocentric Dataset of the University of Barcelona (EDUB) [78], which is divided into different sub–data sets depending on the data annotations, such as EDUB-Obj data set for object localization or segmentation [89], EDUB-Seg data set for egocentric event segmentation [37,38], and EDUB-SegDesc data set that can be used either for egocentric event segmentation or for egocentric sequence description [90].

### Lifelog Retrieval Software Tools

Throughout the referred challenges and workshops, several applications were presented. The Lifelog Search Challenge has been one of the challenges in which several lifelogging systems have been presented with several utilities for real-world use, unlike other challenges, such as ImageCLEFlifelog challenges, which present very specific tasks.

A retrieval and exploration lifelogging system, called lifeXplore, which allows to search and browse features that have been optimized for lifelog data, was presented by Münzer et al [91]. It was based on a video search system, diveXplore [92-94], previously developed for video retrieval competitions. Besides efficient presentation and summarization of lifelog data, it includes different methods of retrieving and visualizing content, such as feature map, day inspector, lifelog filter, sketch search, and similarity search. Over time, the lifeXplore system was improved by including location-based filtering, automatic feature map browsing, and optical character recognition. Moreover, uniform sampling was used as an alternative method for segmenting videos [95,96].

Other tools obtained from video retrieval competitions are the VIRET tool [97-100], which is an updated version of the SIRET interactive video retrieval tool [101] addressing specific properties of visual lifelogs, and vitrivr [102,103], which was developed for video retrieval [104] and later adapted to support multimodal data [105], such as lifelogs.

Zhou et al [106] proposed an iterative lifelog search engine called LIFER, which is queried based on several different forms of lifelog data, such as visual concepts, activities, locations, time, and so on. Despite some limitations of LIFER, this application allows users to retrieve the moments from their personal life archives in a reliable and efficient manner. Enhanced versions of LIFER, such as LIFER 2.0 [107] and LIFER 3.0 [108], were proposed with additional visual features to solve several tasks of ImageCLEFlifelog 2019 and 2020, respectively. It should be noted that many other applications have been proposed in the challenges and workshops mentioned previously [109-114].

In addition to the mentioned applications, other applications have been incorporated into the context of health care. Health lifelogs focus on medical and clinical perspectives. In this case, lifelogs exploit other sensors to gather information. Physical activity, heart rate, blood pressure, and body temperature are examples of measurements that may be valuable from a clinical perspective [61,115,116]. Lifelogs can be used to create platforms that provide a collection of digital memories in a structured and searchable manner, similar to the DigMem system [117]. Another example of an application is the compilation of a diary based on information extracted from the lifelogs [40].

A recent study introduced the use of lifelog monitoring for the early detection of complications in pregnancy [116]. These lifelogs feature physiological data and self-reported information. The authors aimed to detect physiological changes and, together with the multiomics data, try to understand the mechanisms responsible for pregnancy-related diseases. Kim et al [118] proposed the development of a ubiquitous health care system based on biological and lifelog data. This system was designed to assist the care of patients with chronic medical conditions. A Japanese study discussed the viability of a platform (PeOPLe) containing self-recorded lifelogs and medical records to support health care applications [115]. Each patient should provide lifelogs to the platform to assist the health management of the patients who are old and request physician support based on automatic predictions. Similar to PeOPLe, the study presented by Choi et al [61] identified machine learning and mobile learning as helpful tools to examine big data resulting from lifelogs.

In addition to developing diagnostic and health care systems, as illustrated by the examples mentioned previously, lifelogging can assist the change of lifestyle and behaviors [119]. The awareness provided by self-monitoring encourages users to make healthy choices, and if the progression is noticeable, they feel motivated to continue. This applies to nutrition, physical activity, sports, active travel, and psychological well-being [2,40,64,115,120].

### Applications

Lifelogs comprise data of different natures, and consequently, they present an extensive range of possible applications within different use cases, as presented in Table 3. It is noteworthy that work or other procedures may be recorded through lifelogging. An example is the visual lifelogging of a workday by health care professionals [2]. Despite the popularity of wearable lifelogging devices, other sensors can be strategically placed to monitor user activity. These sensors can be used for older people with assisted living needs, and the data acquired by them can be recorded as a lifelog.

In summary, besides memory assistance, monitoring is the main application of lifelogging in health care. This is specifically relevant for the older population, but not exclusively. In addition, monitoring prompts self-reflection by the user, resulting in the motivation for self-improvement.

**Table 3:** Applications of the research presented in the selected publications considering 5 major areas.

Study	Daily activities	Event segmentation	Health care	Summarization	Retrieval
Piasek et al [24]	—^a^	—	✓	—	—
Hodges et al [17]	—	—	✓	—	—
Doherty et al [29]	✓	—	—	—	—
Gurrin et al [30]	✓	—	—	—	—
Pauly-Takacs et al [31]	—	—	✓	—	—
Wang et al [32,33]	✓	—	—	—	—
Song et al [34]	✓	—	—	—	—
Li et al [35]	—	✓	—	—	—
Bolanos et al [36]	—	✓	—	✓	—
Talavera et al [37]	—	✓	—	—	—
Dimiccoli et al [38]	—	✓	—	—	—
Gupta and Gurrin [39]	—	✓	—	—	—
Fan et al [40]	—	—	—	✓	—
Garcia del Molino et al [41]	—	✓	—	—	—
Furnari et al [42]	—	✓	—	—	—
Oliveira-Barra et al [43]	—	—	—	✓	✓
Ellis and Lee [44]	—	✓	—	—	—
Shaikh et al [45]	✓	—	—	—	—
Shah et al [46]	—	—	—	—	✓
Yamano and Itou [47]	—	✓	—	—	—
Ziaei et al [48]	—	✓	—	—	—
Li et al [49]	✓	—	—	—	—
Tanaka et al [50]	✓	—	—	—	—
Doherty et al [54]	✓	—	—	✓	—
Hurvitz et al [55]	✓	—	—	—	—
Yang et al [56,57]	✓	—	✓	—	—
Dobbins et al [58]	✓	—	✓	—	—
Ni et al [59]	✓	—	✓	—	—
Kim et al [60]	—	—	—	—	✓
Choi et al [61]	—	—	✓	—	✓
Dobbins and Fairclough [62]	—	—	✓	—	—
Leibetseder and Schoeffmann [96]	—	—	—	✓	✓
Kovalčík et al [100]	—	—	—	—	✓
Gasser et al [105]	—	—	—	—	✓
Le et al [108]	—	—	—	—	✓
Le et al [110]	—	—	—	—	✓
Ribeiro et al [109]	—	—	—	—	✓
Mai-Nguyen et al [111]	—	✓	—	—	✓
Tran et al [112]	—	✓	—	—	✓
Rossetto et al [113]	—	—	—	—	✓
Khan et al [114]	—	—	—	—	✓
Dobbins et al [117]	—	—	—	—	✓
Karako et al [115]	—	—	✓	—	—
Sugawara et al [116]	—	—	✓	—	—
Kim et al [118]	—	—	✓	—	—
Dobbins and Fairclough [64]	—	—	✓	—	—

^a^The computational tool does not focus on this application.

### Privacy and Concerns

One of the most evident challenges associated with lifelogging is infringement of privacy [2,108]. The nonconsensual logging of bystanders and even the logging of aware friends and family exposes them. A possible solution for visual lifelogs is to blur faces [21,121]. However, when visual lifelogs are used as a memory aid, blurring the faces may hinder this function. Moreover, lifelogs may pose a privacy threat to the surrounding people. Lifeloggers are also susceptible to privacy issues, as lifelogs may constitute valuable information for corporations, including advertisers, which reinforces the necessity of the General Data Protection Regulation. Nevertheless, unobtrusive recording of audio or capturing of images without the explicit consent of everyone involved is prohibited by law.

The use of smartwatches by lifeloggers can be advantageous for recording health data. Kim et al [60] proposed a method to collect data from smartwatches while preserving the user’s privacy. This study is of interest as it attempts to circumvent privacy issues regarding the use of smartwatches. These principles can serve as inspiration for similar approaches for other devices.

Lifelogs may affect our perception of reality; for example, memories may seem more recent than they actually are [119]. Furthermore, despite all the efforts, lifelogs can only capture a small fraction of reality, and as such, only concrete information about subjective experiences can be recorded. Consequently, lifelogs cannot be considered as the ground truth, as there may be failures that prevent full documentation [21].

Another result of our analysis was the permanent character inherent in lifelogs. Although this is advantageous for applications such as memory retrieval, it may become problematic. For example, people with mental illnesses may be obsessed with some memories and dwell on them [7]. Furthermore, even for healthy people, this permanent record may put them under the impression that they are not allowed to change [119]. Therefore, it has been proposed in the literature that lifelogs should try to mimic human memory and implement a forgetting functionality [2,21,64].

Another pertinent concern regarding lifelogging is the possibility that people may rely excessively on lifelogs to remember [119]. This is specifically relevant for future research, as the goal is to enhance the memory of healthy people or improve the memory of people with dementia.

With the popularization of lifelogs and adherence by most of the population, surveillance may become an issue. On one hand, law enforcement may consider lifelogs as a viable method to investigate criminals, which may result in intrusion of the privacy of innocent people [21]. On the other hand, lifelogs may be admitted as proof of innocence. In addition, lifelogs can also potentially empower surveillance by authorities. A legitimate ethical question that emerges from this surveillance is whether illegal behaviors perpetrated by bystanders should be reported by lifeloggers [2].

## Discussion

### Principal Findings

In lifelogging, devices should be ubiquitous, and data capture should occur without requiring any action on the part of the wearer. Currently, everything and everybody with network connectivity can be turned into sensors that continuously generate data. Mobile and wearable devices have been integrated into everyday activities in a seamless and ubiquitous manner. It has become increasingly possible to remotely monitor behaviors using our smartphones or wearable devices.

Lifelogs are personal data created through life experiences and behaviors of individuals during their daily life, such as images, videos, audio, biometric data, or locations, that are collected by physical sensors. Lifelogs are prone to become a powerful tool to retrieve memories or increase the knowledge about an individual’s experiences or behaviors. However, regarding human digital memories (or personal digital memories), different viewpoints arise. Although some refer to human digital memories interchangeably with lifelogs, it is valid to argue that human digital memories are the result of the processing and organization of lifelogs [2,122,123].

Visual lifelogs are one of the most used data in lifelogging approaches and applications. These lifelogs provide important visual information such as environment, objects, activity, and behavior, which are performed and visualized by the lifelogger. As human beings, we can distinguish this visual information and interpret it to reconstruct a memory that was previously experienced. However, for machines such as our computers, this information is only pixels or numbers, which requires the development of algorithms and methods for the interpretation and analysis of these data to retrieve a specific memory efficiently. One of the main advantages of visual lifelogging is the resulting feeling of security. The users are not worried about remembering because they know that everything is being documented [21]. It should be noted that visual lifelogs are usually accompanied by supplementary information, as illustrated by the examples analyzed in the previous sections. These data can help in memory retrieval, because the richer the lifelogs, the more likely they are to hold relevant cues.

Audio lifelogs are less used in lifelogging applications than visual lifelogs because of the additional challenges that they bring to the application. They can be uncomfortable for the lifelogger. However, audio lifelogs may contain important information for lifelogging applications, such as conversations, speeches, music, or several environmental sounds. Moreover, visual entry lifelogs can take advantage of sound records, as illustrated in the cases mentioned in the *Results* section. Although audio devices are mainly used as reminder devices, voice records can be used to document important events as the user is experiencing them or shortly thereafter. However, there is a lack of studies on the use of audio lifelogs and their relevance in lifelogging applications for people with dementia.

Location-based lifelogs allow people to retrieve information about the environment and activities that may occur in that location. Regarding memory retrieval, the locations complemented by other information, such as visual lifelogs or temporal features, facilitate the search for these data and make a lifelogging system more accurate [21]. For example, people with dementia tend to lose their ability to recognize familiar places or locations or become lost and confused about their location. Such information can be retrieved together with visual lifelogs and, therefore, stimulate the memory of these people.

Extracting physical activities only from images is a complex process and sometimes inaccurate, because certain objects or scenes can be associated with a wide range of activities. However, lifelog data such as heart rate and accelerometer data can be used to recognize activities of the lifelogger. By using semantic concepts extracted from the images and locations, the classification of these activities can improve significantly. Human physiological signals have several potential benefits in lifelogging applications, such as for health care and daily life monitoring. However, to use a wide range of these data, several sensors are necessary, and most existing lifelogging technologies do not incorporate all these sensors. For example, multiple devices are required to collect these signals from an individual in real time, which becomes challenging for data synchronization and filtering [64].

Physiological data are rarely used in isolation, and generally, these data alone rarely show cues to retrieve memories, particularly in patients with dementia, as their memories are triggered mainly by visual information. The main utility of physiological data in lifelogging is for medical records and physical activity. However, they may also be used to detect emotions, and similar to visual lifelogs, they can form a more complete digital memory [117].

Regarding privacy and concerns, lifeloggers must have access to their data and opportunities to rectify, remove, and control the data that is collected. In addition, lifeloggers should be aware of how their data are stored and used, who owns the lifelogs, and who owns the information obtained from their lifelogs [119]. Gurrin et al [2] assume that the data gatherer owns the lifelogs, which raises the question, “What happens to lifelogs when the correspondent lifelogger dies?” On one hand, lifelogs contain a lifetime of personal information. However, if they are stored in databases, it can help to improve research approaches. Thus, it is necessary to establish regulations on how to approach these concerning issues.

### Conclusions

The integration of lifelogging into people’s lives can be beneficial to improve the quality of their life, either by serving as a tool for memory augmentation or by providing support when having memory issues. Lifelogging systems can create relevant digital memories. Through this narrative review, we understand that contextual information can be extracted from lifelogs, which provides an understanding of a person’s daily activities based on events, experiences, and behaviors.

Initially, the scientific community in the lifelogging research field focused their attention on the design and development of solutions or devices capable of acquiring and storing data without interfering with one’s daily life. However, with the increase in wearable devices available for personal data acquisition and the large amount of data to be stored and retrieved, new challenges and issues arose regarding the storage, processing, organization, and retrieval of lifelogs.

An important conclusion of this research exercise is that visual lifelogs are most prevalent when the goal is to create digital memories as surrogate memories. Nevertheless, there is a tendency to associate visual lifelogs with other lifelog data such as audio, location, physical activities, and physiological signals. Audio lifelogs can provide relevant information, such as speeches or environmental sounds, which encode information about locations, activities, and overall context. Along with these personal data, location-based lifelogs can provide additional information. Physical activity and physiological lifelog data are often associated with health care and quality of life. The several sensors that can be incorporated in wearable and easy-to-use devices provide useful information for the recognition and classification of the activities and behaviors of a user. These data used in isolation have some benefits for health care and personal monitoring. Nevertheless, when combined with other lifelogs, they potentially provide important cues to retrieve and form more complete personal digital memories. In addition to creating human digital memories, the acquisition and processing of these lifelogs can be used for monitoring daily life and self-improvement. As they comprise data of different natures, they present an extensive range of possible applications within different use cases. In addition to their relevance in health care, several other applications have been explored such as daily activity analysis, event segmentation, summarization, and information retrieval.

The practice of lifelogging requires tracking and recording of lifelogs in everyday life, for which it is necessary to capture personal data over long periods or even the lifelogger’s entire life. These lifelogs can be combined to develop methods to recognize several contextual data to provide a broader understanding of the lifelogger’s life, such as events, experiences, behaviors, and moments. However, the lifelogs must be synchronized with each other, which can be achieved through time features recorded at the time of lifelog acquisition.

Nevertheless, when these lifelogs are introduced into a lifelogging application, some of them are not relevant or do not contain useful information for further processing and visualization. Therefore, preprocessing methods can be applied to select only relevant lifelogs and remove or correct those that may introduce errors and noise into the system. To retrieve and visualize the previously selected lifelogs, the lifelogging system must be able to interpret these lifelogs in a way similar to that of the lifelogger. Therefore, it is important to annotate, organize, and store the lifelogs with semantic concepts that provide more information about the environment and activities of the lifelogger. These semantic concepts are useful to understand the lifelogger’s behavior and define events and specific moments, which may be required and visualized in the future as surrogate memories.

This narrative review shows that there is a considerable number of published studies on lifelogging. However, we identified several open questions through the analysis and possible lines of investigation in this currently important topic.

## Abbreviations

EDUB: Egocentric Dataset of the University of Barcelona
NTCIR: National Institute of Informatics Testbeds and Community for Information Access Research
PRISMA: Preferred Reporting Items for Systematic Reviews and Meta-Analyses

## References

[1] Dodge M, Kitchin R (2016). ‘Outlines of a world coming into existence’: pervasive computing and the ethics of forgetting. Environ Plann B Plann Des.

[2] Gurrin C, Smeaton AF, Doherty AR (2014). LifeLogging: personal big data. FNT Inf Retrieval.

[3] Bush V (1996). As we may think. Interactions.

[4] Mann S (1997). Wearable computing: a first step toward personal imaging. Computer.

[5] Mann S (1998). 'WearCam' (The wearable camera): personal imaging systems for long-term use in wearable tetherless computer-mediated reality and personal photo/videographic memory prosthesis. Proceedings of the Digest of Papers. Second International Symposium on Wearable Computers (Cat. No.98EX215).

[6] Mann S (2004). Continuous lifelong capture of personal experience with EyeTap. Proceedings of the the 1st ACM workshop on Continuous archival and retrieval of personal experiences.

[7] Mann S, Fung J, Aimone C, Sehgal A, Chen D (2005). Designing EyeTap digital eyeglasses for continuous lifelong capture and sharing of personal experiences.

[8] Mann S, Huang J, Janzen R, Lo R, Rampersad V, Chen A, Doha T (2011). Blind navigation with a wearable range camera and vibrotactile helmet. Proceedings of the 19th ACM international conference on Multimedia.

[9] Mann S, Lo R, Ovtcharov K, Gu S, Dai D, Ngan C, Ai T (2012). Realtime HDR (High Dynamic Range) video for eyetap wearable computers, FPGA-based seeing aids, and glasseyes (EyeTaps). Proceedings of the 2012 25th IEEE Canadian Conference on Electrical and Computer Engineering (CCECE).

[10] Mann S, Ali M, Lo R, Wu H (2013). FreeGlass for developers, “haccessibility”, and Digital Eye Glass + Lifeglogging research in a (sur/sous)veillance society. Proceedings of the International Conference on Information Society (i-Society 2013).

[11] Mann S, Mann C, Lam D, Mathewson K, Stairs J, Pierce C, Hernandez J, Kanaan G, Piette L, Khokhar H (2019). The human eye as a camera. Proceedings of the 2019 IEEE International Conference on E-health Networking, Application & Services (HealthCom).

[12] Gemmell J, Bell G, Lueder R, Drucker S, Wong C (2002). Mylifebits: fulfilling the memex vision. Proceedings of the tenth ACM international conference on Multimedia.

[13] Gemmell J, Lueder R, Bell G (2003). The mylifebits lifetime store. Proceedings of the 2003 ACM SIGMM workshop on Experiential telepresence.

[14] Gemmell J, Bell G, Lueder R (2006). MyLifeBits. Commun ACM.

[15] Bell G, Gemmell J (2007). A digital life. Sci Am.

[16] Bell G, Gemmell J (2009). Total Recall: How the E-Memory Revolution Will Change Everything.

[17] Hodges S, Williams L, Berry E, Izadi S, Srinivasan J, Butler A, Smyth G, Kapur N, Wood K, Dourish P, Friday A (2006). SenseCam: a retrospective memory aid. UbiComp 2006: Ubiquitous Computing. UbiComp 2006. Lecture Notes in Computer Science, vol 4206.

[18] Hodges S, Berry E, Wood K (2011). SenseCam: a wearable camera that stimulates and rehabilitates autobiographical memory. Memory.

[19] Chen M, Mao S, Liu Y (2014). Big data: a survey. Mobile Netw Appl.

[20] Gurrin C, Smeaton AF, Byrne D, O'Hare N, Jones GJF, O'Connor N, Li H, Liu T, Ma WY, Sakai T, Wong KF, Zhou G (2008). An examination of a large visual lifelog. Information Retrieval Technology. AIRS 2008: Lecture Notes in Computer Science, vol 4993.

[21] Harvey M, Langheinrich M, Ward G (2016). Remembering through lifelogging: a survey of human memory augmentation. Pervasive Mobile Comput.

[22] (2019). World Population Ageing 2017 Highlights.

[23] (2017). Global Action Plan on the Public Health Response to Dementia 2017 - 2025.

[24] Piasek P, Irving K, Smeaton AF (2012). Case study in SenseCam use as an intervention technology for early-stage dementia. Int J Comput Healthcare.

[25] Ferrari R (2015). Writing narrative style literature reviews. Med Writing.

[26] Greenhalgh T, Thorne S, Malterud K (2018). Time to challenge the spurious hierarchy of systematic over narrative reviews?. Eur J Clin Invest.

[27] Page M, McKenzie J, Bossuyt P, Boutron I, Hoffmann T, Mulrow C, Shamseer L, Tetzlaff JM, Moher D (2021). Updating guidance for reporting systematic reviews: development of the PRISMA 2020 statement. J Clin Epidemiol.

[28] Doherty AR, Smeaton AF (2008). Automatically segmenting lifelog data into events. Proceedings of the 2008 Ninth International Workshop on Image Analysis for Multimedia Interactive Services.

[29] Doherty AR, Caprani N, Conaire CO, Kalnikaite V, Gurrin C, Smeaton AF, O’Connor NE (2011). Passively recognising human activities through lifelogging. Comput Human Behav.

[30] Gurrin C, Qiu Z, Hughes M, Caprani N, Doherty AR, Hodges SE, Smeaton AF (2013). The smartphone as a platform for wearable cameras in health research. Am J Prev Med.

[31] Pauly-Takacs K, Moulin CJ, Estlin EJ (2011). SenseCam as a rehabilitation tool in a child with anterograde amnesia. Memory.

[32] Wang P, Smeaton AF (2013). Using visual lifelogs to automatically characterize everyday activities. Inf Sci.

[33] Wang P, Sun L, Yang S, F. Smeaton A, Gurrin C (2016). Characterizing everyday activities from visual lifelogs based on enhancing concept representation. Comput Vision Image Understanding.

[34] Song S, Chandrasekhar V, Cheung N, Narayan S, Li L, Lim J (2015). Activity recognition in egocentric life-logging videos. Computer Vision - ACCV 2014 Workshops.

[35] Li N, Crane M, Ruskin HJ, Gurrin C (2013). Application of statistical physics for the identification of important events in visual lifelogs. Proceedings of the 2013 IEEE International Conference on Bioinformatics and Biomedicine.

[36] Balonas M, Mestre B, Talavera E, Giró-i-Nieto X, Radeva P (2015). Visual summary of egocentric photostreams by representative keyframes. Proceedings of the 2015 IEEE International Conference on Multimedia & Expo Workshops (ICMEW).

[37] Talavera E, Bolanos M, Dimiccoli M, Aghaei M, Radeva P (2015). R-clustering for egocentric video segmentation. Pattern Recognition and Image Analysis.

[38] Dimiccoli M, Bolaños M, Talavera E, Aghaei M, Nikolov SG, Radeva P (2017). SR-clustering: semantic regularized clustering for egocentric photo streams segmentation. Comput Vision Image Understanding.

[39] Gupta R, Gurrin C (2018). Approaches for event segmentation of visual lifelog data. MultiMedia Modeling. MMM 2018. Lecture Notes in Computer Science, vol 10704.

[40] Fan C, Zhang Z, Crandall DJ (2018). Deepdiary: lifelogging image captioning and summarization. J Visual Commun Image Representation.

[41] Garcia del Molino A, Lim J-H, Tan A-H (2018). Predicting visual context for unsupervised event segmentation in continuous photostreams. Proceedings of the 26th ACM international conference on Multimedia.

[42] Furnari A, Battiato S, Farinella GM (2018). Personal-location-based temporal segmentation of egocentric videos for lifelogging applications. J Visual Commun Image Representation.

[43] Oliveira-Barra G, Bolanos M, Talavera E, Gelonch O, Garolera M, Radeva P, Alameda-Pineda X, Ricci E, Sebe N (2019). Lifelog retrieval for memory stimulation of people with memory impairment. Multimodal Behavior Analysis in the Wild: Advances and Challenges.

[44] Ellis D, Lee K (2004). Minimal-impact audio-based personal archives. Proceedings of the the 1st ACM workshop on Continuous archival and retrieval of personal experiences.

[45] Shaikh MA, Molla MK, Hirose K (2008). Automatic life-logging: a novel approach to sense real-world activities by environmental sound cues and common sense. Proceedings of the 2008 11th International Conference on Computer and Information Technology.

[46] Shah M, Mears B, Chakraborty C, Spanias A (2012). Lifelogging: archival and retrieval of continuously recorded audio using wearable devices. Proceedings of the 2012 IEEE International Conference on Emerging Signal Processing Applications.

[47] Yamano K, Itou K (2009). Browsing audio lifelog data using acoustic and location information. Proceedings of the 2009 Third International Conference on Mobile Ubiquitous Computing, Systems, Services and Technologies.

[48] Ziaei A, Sangwan A, Hansen JH (2013). Prof-life-log: Personal interaction analysis for naturalistic audio streams. Proceedings of the 2013 IEEE International Conference on Acoustics, Speech and Signal Processing.

[49] Li D, Gu Y, Kamijo S (2018). Smartphone based lifelog with meaningful place detection. Proceedings of the 2018 IEEE International Conference on Consumer Electronics (ICCE).

[50] Tanaka G, Okada M, Mineno H (2015). GPS-based daily context recognition for lifelog generation using smartphone. Int J Advanced Comput Sci App.

[51] Aizawa K, Ishijima K, Shiina M (2001). Summarizing wearable video. Proceedings 2001 International Conference on Image Processing (Cat. No.01CH37205).

[52] Hori T, Aizawa K (2003). Context-based video retrieval system for the life-log applications. Proceedings of the 5th ACM SIGMM international workshop on Multimedia information retrieval.

[53] Datchakorn T, Toshihiko Y, Kiyoharu A (2005). Practical experience recording and indexing of life log video. Proceedings of the 2nd ACM workshop on Continuous archival and retrieval of personal experiences.

[54] Doherty AR, Kelly P, Kerr J, Marshall S, Oliver M, Badland H, Hamilton A, Foster C (2013). Using wearable cameras to categorise type and context of accelerometer-identified episodes of physical activity. Int J Behav Nutr Phys Act.

[55] Hurvitz PM, Moudon AV, Kang B, Saelens BE, Duncan GE (2014). Emerging technologies for assessing physical activity behaviors in space and time. Front Public Health.

[56] Yang P, Hanneghan M, Qi J, Deng Z, Dong F, Fan D (2015). Improving the validity of lifelogging physical activity measures in an internet of things environment. Proceedings of the 2015 IEEE International Conference on Computer and Information Technology; Ubiquitous Computing and Communications; Dependable, Autonomic and Secure Computing; Pervasive Intelligence and Computing.

[57] Yang P, Stankevicius D, Marozas V, Deng Z, Liu E, Lukosevicius A, Dong F, Xu L, Min G (2018). Lifelogging data validation model for internet of things enabled personalized healthcare. IEEE Trans Syst Man Cybern Syst.

[58] Dobbins C, Rawassizadeh R, Momeni E (2017). Detecting physical activity within lifelogs towards preventing obesity and aiding ambient assisted living. Neurocomputing.

[59] Ni J, Chen B, Allinson NM, Ye X (2020). A hybrid model for predicting human physical activity status from lifelogging data. Eur J Operational Res.

[60] Kim JW, Lim JH, Moon SM, Jang B (2019). Collecting health lifelog data from smartwatch users in a privacy-preserving manner. IEEE Trans Consum Electron.

[61] Choi J, Choi C, Ko H, Kim P (2016). Intelligent healthcare service using health lifelog analysis. J Med Syst.

[62] Dobbins C, Fairclough S (2019). Signal processing of multimodal mobile lifelogging data towards detecting stress in real-world driving. IEEE Trans Mobile Comput.

[63] Doherty A, Pauly-Takacs K, Caprani N, Gurrin C, Moulin CJ, O'Connor N, Smeaton AF (2012). Experiences of aiding autobiographical memory using the SenseCam. Human Comput Interact.

[64] Dobbins C, Fairclough S (2016). Lifelogging technologies to detect negative emotions associated with cardiovascular disease. Applied Computing in Medicine and Health.

[65] Lee H, Smeaton AF, O’Connor NE, Jones G, Blighe M, Byrne D, Doherty A, Gurrin C (2008). Constructing a SenseCam visual diary as a media process. Multimedia Syst.

[66] Gibson G, Newton L, Pritchard G, Finch T, Brittain K, Robinson L (2016). The provision of assistive technology products and services for people with dementia in the United Kingdom. Dementia (London).

[67] Sanders D, Scott P (2020). Literature review: technological interventions and their impact on quality of life for people living with dementia. BMJ Health Care Inform.

[68] Rodgers MM, Pai VM, Conroy RS (2015). Recent advances in wearable sensors for health monitoring. IEEE Sensors J.

[69] Zheng Y, Ding X, Poon CC, Lo BP, Zhang H, Zhou X, Yang G, Zhao N, Zhang Y (2014). Unobtrusive sensing and wearable devices for health informatics. IEEE Trans Biomed Eng.

[70] Gemmell J, Sundaram H (2004). CARPE'04: Proceedings of the the 1st ACM workshop on Continuous archival and retrieval of personal experiences.

[71] Gemmell J, Sundaram H (2005). CARPE '05: Proceedings of the 2nd ACM workshop on Continuous archival and retrieval of personal experiences.

[72] Mase K (2006). CARPE '06: Proceedings of the 3rd ACM workshop on Continuous archival and retrival of personal experences.

[73] GemmellJ G, Williams L, Wood K, Lueder R, Bell G (2004). Passive capture and ensuing issues for a personal lifetime store. Proceedings of the the 1st ACM workshop on Continuous archival and retrieval of personal experiences.

[74] Aizawa K, Tancharoen D, Kawasaki S, Yamasaki T (2004). Efficient retrieval of life log based on context and content. Proceedings of the the 1st ACM workshop on Continuous archival and retrieval of personal experiences.

[75] Gurrin C, Joho H, Hopfgartner F, Zhou L, Albatal R (2016). NTCIR lifelog: the first test collection for lifelog research. Proceedings of the 39th International ACM SIGIR conference on Research and Development in Information Retrieval.

[76] Gurrin C, Joho H, Hopfgartner F, Zhou L, Gupta R, Albatal R, Dang-Nguyen D-T (2017). Overview of NTCIR-13 Lifelog-2 Task. Proceedings of the The Thirteenth NTCIR conference (NTCIR-13).

[77] Gurrin C, Joho H, Hopfgartner F, Zhou L, Ninh H-T, Le T-K, Albatal R, Dang-Nguyen D-T, Healy G (2019). Overview of the NTCIR-14 Lifelog-3 task. Proceedings of the 14th NTCIR Conference on Evaluation of Information Access Technologies.

[78] Bolanos M, Dimiccoli M, Radeva P (2017). Toward storytelling from visual lifelogging: an overview. IEEE Trans Human Mach Syst.

[79] Gurrin C, Joho H, Hopfgartner F, Zhou L, Albatal R (2016). Overview of NTCIR-12 lifelog task. Proceedings of the 12th NTCIR Conference on Evaluation of Information Access Technologies.

[80] Gurrin C, Giro-i-Nieto X, Radeva P, Dimiccoli M, Johansen H, Joho H, Singh VK (2016). LTA 2016: the first workshop on lifelogging tools and applications. Proceedings of the 24th ACM international conference on Multimedia.

[81] Dang-Nguyen D, Piras P, Riegler M, Boato G, Zhou L, Gurrin C (2017). Overview of ImageCLEF lifelog 2017: lifelog retrieval and summarization. ImageCLEF.

[82] Dang-Nguyen DT, Piras L, Riegler M, Zhou L, Lux M, Gurrin C (2018). Overview of ImageCLEFlifelog 2018: daily living understanding and lifelog moment retrieval. Proceedings of the Conference and Labs of the Evaluation Forum.

[83] Dang-Nguyen DT, Piras L, Riegler M, Zhou L, Lux M, Tran M-T, Le T-K, Ninh V-T, Gurrin C (2019). Overview of ImageCLEFlifelog 2019: solve my life puzzle and lifelog moment retrieval. Proceedings of CLEF 2019.

[84] Ninh V-T, Le T-K, Zhou L, Piras L, Riegler M, Halvorsen P, Lux M, Tran M-T, Gurrin C, Dang-Nguyen D-T (2020). Overview of ImageCLEF Lifelog 2020: lifelog moment retrieval and sport performance lifelog. Proceedings of CLEF 2020.

[85] Gurrin C, Schoeffmann K, Joho H, Dang-Nguyen DT, Riegler M, Piras L (2018). LSC '18: Proceedings of the 2018 ACM Workshop on The Lifelog Search Challenge.

[86] Gurrin C, Schoeffmann K, Joho H, Dang-Nguyen DT, Riegler M, Piras L (2019). LSC '19: Proceedings of the ACM Workshop on Lifelog Search Challenge.

[87] Gurrin C, Schoeffmann K, Jónsson BÞ, Dang-Nguyen DT, Lokoč J, Tran MT, Hürst W (2020). LSC '20: Proceedings of the Third Annual Workshop on Lifelog Search Challenge.

[88] Rawassizadeh R, Tomitsch M, Wac K, Tjoa AM (2012). UbiqLog: a generic mobile phone-based life-log framework. Pers Ubiquit Comput.

[89] Bolaños M, Radeva P (2015). Ego-object discovery. arXiv.

[90] Bolaños M, Peris A, Casacuberta F, Soler S, Radeva P (2018). Egocentric video description based on temporally-linked sequences. J Visual Commun Image Representation.

[91] Münzer B, Leibetseder A, Kletz S, Primus MJ, Schoeffmann K (2018). lifeXplore at the lifelog search challenge 2018. Proceedings of the 2018 ACM Workshop on The Lifelog Search Challenge.

[92] Schoeffmann K, Primus MJ, Muenzer B, Petscharnig S, Karisch C, Qing X, Huerst W, Amsaleg L, Guðmundsson G, Gurrin C, Jónsson B, Satoh S (2017). Collaborative feature maps for interactive video search. MultiMedia Modeling. MMM 2017. Lecture Notes in Computer Science, vol 10133.

[93] Primus MJ, Münzer B, Leibetseder A, Schoeffmann K (2018). The ITEC collaborative video search system at the video browser showdown 2018. MultiMedia Modeling. MMM 2018. Lecture Notes in Computer Science, vol 10705.

[94] Leibetseder A, Münzer B, Primus MJ, Kletz S, Schoeffmann K (2020). diveXplore 4.0: the ITEC deep interactive video exploration system at VBS 2020. MultiMedia Modeling.

[95] Leibetseder A, Münzer B, Primus MJ, Kletz S, Schoeffmann K, Berns F, Beecks C (2019). Lifexplore at the lifelog search challenge 2019. Proceedings of the ACM Workshop on Lifelog Search Challenge.

[96] Leibetseder A, Schoeffmann K (2020). Lifexplore at the lifelog search challenge 2020. Proceedings of the Third Annual Workshop on Lifelog Search Challenge.

[97] Lokoč J, Souček T, Kovalčik G (2018). Using an interactive video retrieval tool for lifelog data. Proceedings of the 2018 ACM Workshop on The Lifelog Search Challenge.

[98] Lokoč J, Kovalčík G, Souček T, Moravec J, Čech P (2019). Viret: a video retrieval tool for interactive known-item search. Proceedings of the 2019 on International Conference on Multimedia Retrieval.

[99] Lokoč J, Souček T, Čech P, Kovalčík G (2019). Enhanced VIRET tool for lifelog data. Proceedings of the ACM Workshop on Lifelog Search Challenge.

[100] Kovalčík G, Škrhak V, Souček T, Lokoč J (2020). VIRET tool with advanced visual browsing and feedback. Proceedings of the Third Annual Workshop on Lifelog Search Challenge.

[101] Lokoč J, Kovalcík G, Soucek T (2018). Revisiting SIRET video retrieval tool. International Conference on Multimedia Modeling.

[102] Rossetto L, Gasser R, Heller S, Parian M, Schuldt H (2019). Retrieval of structured and unstructured data with vitrivr. Proceedings of the ACM Workshop on Lifelog Search Challenge.

[103] Heller S, Parian M, Gasser R, Sauter L, Schuldt H (2020). Interactive lifelog retrieval with vitrivr. Proceedings of the Third Annual Workshop on Lifelog Search Challenge.

[104] Rossetto L, Giangreco I, Tanase C, Schuldt H (2016). Vitrivr: a flexible retrieval stack supporting multiple query modes for searching in multimedia collections. Proceedings of the 24th ACM international conference on Multimedia.

[105] Gasser R, Rossetto L, Schuldt H (2019). Multimodal multimedia retrieval with vitrivr. Proceedings of the 2019 on International Conference on Multimedia Retrieval.

[106] Zhou L, Hinbarji Z, Dang-Nguyen D-T, Gurrin C (2018). Lifer: an interactive lifelog retrieval system. Proceedings of the 2018 ACM Workshop on The Lifelog Search Challenge.

[107] Ninh V-T, Le T-K, Zhou L, Piras L, Riegler M, Lux M, Tran M-T, Gurrin C, Dang-Nguyen D (2019). LIFER 2.0: discovering personal lifelog insights using an interactive lifelog retrieval system. Proceedings of the CLEF 2019.

[108] Le T-K, Ninh V-T, Zhou L, Nguyen-Ngoc M-H, Trinh H-D, Tran N-H, Piras L, Riegler M, Halvorsen P, Lux M, Tran M-T, Healy G, Gurrin C, Dang-Nguyen D-T (2020). Organiser team at ImageCLEF Lifelog 2020: a baseline approach for moment retrieval and athlete performance prediction using lifelog data. Proceedings of the CLEF 2020.

[109] Ribeiro R, Silva J, Trifan A, Oliveira JL, Neves AJ (2020). UA.PT Bioinformatics at ImageCLEF 2020: lifelog moment retrieval web based tool. Proceedings of the CLEF 2020.

[110] Le T-K, Ninh V-T, Tran M-T, Nguyen T-A, Nguyen H-D, Zhou L, Healy G, Gurrin C (2020). LifeSeeker 2.0: interactive lifelog search engine at LSC 2020. Proceedings of the Third Annual Workshop on Lifelog Search Challenge.

[111] Mai-Nguyen A-V, Phan T-D, Vo A-K, Tran V-L, Dao M-S, Zettsu K (2020). BIDAL-HCMUS@LSC2020: an interactive multimodal lifelog retrieval with query-to-sample attention-based search engine. Proceedings of the Third Annual Workshop on Lifelog Search Challenge.

[112] Tran M-T, Nguyen T-A, Tran Q-C, Tran M-K, Nguyen K, Ninh V-T, Le T-K, Trang-Trung H-P, Le H-A, Nguyen HD (2020). FIRST - Flexible Interactive Retrieval SysTem for visual lifelog exploration at LSC 2020. Proceedings of the Third Annual Workshop on Lifelog Search Challenge.

[113] Rossetto L, Baumgartner M, Ashena N, Ruosch F, Pernischová R, Bernstein A (2020). LifeGraph: a knowledge graph for lifelogs. Proceedings of the Third Annual Workshop on Lifelog Search Challenge.

[114] Khan OS, Larsen MD, Poulsen LA, Jónsson B, Zahálka J, Rudinac S, Koelma D, Worring M (2020). Exquisitor at the lifelog search challenge 2020. Proceedings of the Third Annual Workshop on Lifelog Search Challenge.

[115] Karako K, Chen Y, Song P, Tang W (2019). Super-aged society: constructing an integrated information platform of self-recording lifelogs and medical records to support health care in Japan. Biosci Trends.

[116] Sugawara J, Ochi D, Yamashita R, Yamauchi T, Saigusa D, Wagata M, Obara T, Ishikuro M, Tsunemoto Y, Harada Y, Shibata T, Mimori T, Kawashima J, Katsuoka F, Igarashi-Takai T, Ogishima S, Metoki H, Hashizume H, Fuse N, Minegishi N, Koshiba S, Tanabe O, Kuriyama S, Kinoshita K, Kure S, Yaegashi N, Yamamoto M, Hiyama S, Nagasaki M (2019). Maternity Log study: a longitudinal lifelog monitoring and multiomics analysis for the early prediction of complicated pregnancy. BMJ Open.

[117] Dobbins C, Merabti M, Fergus P, Llewellyn-Jones D, Bouhafs F (2013). Exploiting linked data to create rich human digital memories. Comput Commun.

[118] Kim S, Yeom S, Kwon O, Shin D, Shin D (2018). Ubiquitous healthcare system for analysis of chronic patients’ biological and lifelog data. IEEE Access.

[119] Jacquemard T, Novitzky P, O'Brolcháin F, Smeaton AF, Gordijn B (2014). Challenges and opportunities of lifelog technologies: a literature review and critical analysis. Sci Eng Ethics.

[120] Doherty AR, Hodges SE, King AC, Smeaton AF, Berry E, Moulin CJ, Lindley S, Kelly P, Foster C (2013). Wearable cameras in health: the state of the art and future possibilities. Am J Prev Med.

[121] Florez-Revuelta F, Mihailidis A, Ziefle M, Colonna L, Spinsante S (2018). Privacy-aware and acceptable lifelogging services for older and frail people: The PAAL project. Proceedings of the 2018 IEEE 8th International Conference on Consumer Electronics - Berlin (ICCE-Berlin).

[122] Sellen AJ, Whittaker S (2010). Beyond total capture. Commun ACM.

[123] Dobbins C, Merabti M, Fergus P, Llewellyn-Jones D (2014). Creating human digital memories with the aid of pervasive mobile devices. Pervasive Mobile Comput.

